# Synergistic Integration of Multimodal Lasers and Bioactive Substances for Severe Post-traumatic Facial Sequelae and Traumatic Tattooing: A Case Report

**DOI:** 10.7759/cureus.102135

**Published:** 2026-01-23

**Authors:** Aleksandra Szlachcic, Bartosz Szlachcic, Daniela Martinelli, Alessandra Zevini, Riccardo Barini

**Affiliations:** 1 Physiology, Faculty of Medicine, Jagiellonian University Medical College, Kraków, POL; 2 Dermatology, Medistica. Medycyna + Piękno, Kraków, POL; 3 Medical Affairs, Quanta System, Samarate, ITA

**Keywords:** case report, nd:yag laser, picosecond laser, prp injection, traumatic tattoo

## Abstract

Traumatic tattoos (TTs) with chronic scarring present a complex dermatological challenge, often requiring protocols to optimize pigment clearance and skin texture. This case report evaluates the synergy between a picosecond laser (Nd:YAG 1064 nm), a fractional CO₂ laser, and biostimulants. A 33-year-old female presented with a facial TT and scarring resulting from a 10-year-old injury. The lesions were located on the right forehead and the periocular area, the latter involving an underlying titanium plate. Management began with a fractional CO₂ laser combined with platelet-rich plasma (PRP) for initial scar remodeling, followed by a picosecond laser for deep pigment removal and scar refinement. Tropocollagen injections concluded the treatment. Results after six months showed appreciable clinical regression and scar blurring. However, the periocular area demonstrated greater challenges, including prolonged erythema and swelling, due to thermal interaction with the metallic implant. This case report provides valuable data on an effective and safe alternating laser treatment regimen for complex TTs while simultaneously highlighting the necessity for meticulous parameter selection and intensive post-treatment care when dealing with underlying metallic structures.

## Introduction

Traumatic tattoos (TT) represent a significant challenge in dermatology, as they not only negatively alter an individual’s appearance due to the deposition of exogenous pigmented particles (such as asphalt, dust, metal, carbon, or gunpowder) deep within the dermis [1], but are also inherently linked to tissue damage and scarring resulting from the traumatic event, frequently stemming from road traffic accidents.

Since this type of embedding becomes permanent after the wound has fully re-epithelialized, the most effective course of action is prevention. This involves the immediate removal of the particles, ideally before the healing process begins. Thorough scrubbing and mechanical cleaning of the injury are critical steps to avert TT. Once the skin has completely closed, a variety of treatment methods become available [2,3]. The management of these complex lesions requires a therapeutic approach that simultaneously aims for effective pigment removal and improvement of the associated scar quality [1,4].

In modern dermatology, nonablative laser therapy has superseded traditional methods such as surgical excision or dermabrasion, which carry high risks of further scarring. Historically, nanosecond Q-switched (QS) lasers were the gold standard; however, their reliance on photothermal effects can be limiting when treating resistant pigmentation or compromised skin [4].

The emergence of picosecond (Pico) technology represents a significant advancement. By utilizing ultrashort pulse durations (300-900 ps), picosecond lasers favor photoacoustic disruption of pigment over thermal heating, thereby enhancing target specificity and minimizing collateral damage to the surrounding dermis. Despite these advancements, monotherapy often encounters significant limitations when addressing complex traumatic lesions. The intrinsic challenge lies in the divergent therapeutic targets: a wavelength or emission mode optimized for the remodeling of superficial fibrotic tissue typically lacks the penetration depth or peak power required to fragment pigment particles sequestered in the deep dermis. Conversely, settings effective for deep pigment clearance may not provide the necessary thermal or mechanical stimulus for surface texture normalization.

To overcome this therapeutic gap, this article evaluates a sequenced multimodal approach tailored for a challenging traumatic facial tattoo, further complicated by the presence of an underlying titanium orbital implant. The regimen was strategically designed to leverage the synergy between different technologies: a fractional CO₂ laser combined with platelet-rich plasma (PRP) for foundational scar remodeling, followed by targeted pigment removal and additional scar refinement using a nonablative Pico Nd:YAG 1064 nm laser (in both nonfractional mode for deep dyschromia and fractional mode for associated scars), and concluded with the administration of tropocollagen to further support dermal repair. By integrating ablative, picosecond, and bioactive modalities, this combined protocol aims to optimize both structural and aesthetic outcomes, addressing the multifaceted nature of traumatic injuries more effectively than conventional single-device treatments.

## Case presentation

The patient was a 33-year-old female with a Fitzpatrick II skin type who presented as generally healthy, with no reported complaints or residual symptoms, medication use, or allergies at the time of the initial evaluation. Ten years prior, she sustained extensive trauma to the right side of her head and face following a motorcycle accident.

At her first visit, irregular, horizontal, white scars were noted on the right side of the forehead, temple, and around the right eye. The skin surrounding the right eye was blackened due to exogenous particles embedded at various depths; the distribution was mixed, involving both the dermis and epidermis but localized primarily within the epidermis. This finding is consistent with contact with the ground during the accident (likely asphalt, soil, etc.). The skin across the forehead and periorbital area appeared markedly thin, bordering on atrophy.

The injury had caused destruction of the right orbital bones, which had been replaced with a titanium plate. The screws securing this plate were palpable beneath the skin surface. Six years before the examination, the patient had undergone three fractional CO₂ laser ablation treatments targeting the forehead skin. The eye area had not been treated. The patient could not recall the specific parameters or the laser handpiece used during those procedures.

Treatment protocol

The management of this patient, presenting with complex and severe sequelae from extensive facial trauma, necessitated a carefully orchestrated, multiphase protocol. The primary challenge was the need for aggressive dermal remodeling to address profound scarring and marked atrophy while simultaneously ensuring the safety of the extremely thin skin overlying a metallic titanium implant.

To navigate these complexities, energy selections were initially guided by standard clinical protocols and subsequently validated via meticulous test spots. This process ensured that the therapy was perfectly tailored to the patient’s unique tissue response (Table 1).

**Table 1:** Summary of clinical parameters PRP, platelet-rich plasma

Treatment area	Device/technology	Parameters	Bioactive support	Sessions	Interval
Forehead (ablative phase)	Ablative fractional CO₂	15 W, 40 mJ/cm², stack 2, 2 passes	PRP	4	Six to eight weeks
Forehead (consolidation)	Non-ablative Nd:YAG	0.8-1 J/cm², 8 mm spot, 5-10 Hz	Tropocollagen	4	Six to eight weeks
Right eye	Nd:YAG 1064 nm (PS)	1.5-2.0 J/cm², 3 mm spot, 1-5 Hz	-	3	Eight to 10 weeks
Right eye	Fractional Nd:YAG 1064 nm	0.5-0.8 J/cm², 8 mm spot, 5 Hz	-	3	Six to eight weeks

Forehead treatment

The initial approach for the forehead involved four sessions using an ablative fractional CO₂ laser (using a LiteScan handpiece). This choice was deliberate, as the CO₂ laser is the gold standard for deep resurfacing and scar revision. Its action creates microscopic thermal zones, vaporizing damaged tissue and triggering a powerful wound-healing response essential for neocollagenesis. The laser parameters were set at 15 W (40 mJ/cm²), with a pulse density of 3 and a stack of 2, performing two passes per session.

This ablative phase was synergistically combined with PRP injections administered directly into the laser microchannels. This “open-channel” technique ensures deep penetration of growth factors, directly combating skin atrophy in order to accelerate healing, mitigate complication risks, and markedly potentiate collagen and elastin synthesis. Sessions were scheduled six to eight weeks apart.

Following this intensive ablative phase, the treatment transitioned into a consolidation period utilizing a nonablative Nd:YAG 1064 nm laser (Discovery Pico fractional laser, Quanta System SpA, Samarate, Italy) for four additional sessions. This shift was designed to continue stimulating deeper collagen remodeling and improve skin texture without causing further surface damage or extending downtime.

This phase consisted of four additional treatments, spaced six to eight hours apart. Using the Nd:YAG 1064 nm wavelength with the Plasmafrax handpiece (8DF, 8 mm fractional spot), the fluence ranged from 0.8 to 1 J/cm², pulsed at 5-10 Hz, with two to three passes per session. To further bolster the regenerative effort, the patient received supplementary injections of Guna tropocollagen at four-week intervals, which provide the necessary biological scaffolding and precursors to ensure the mature organization of newly formed collagen fibers. A summary of the treatments performed is provided in Table 1.

Right eye area treatment

The strategy for the right eye area was distinct and more specialized due to the delicate nature of the periorbital skin and the specific issues of TT and scarring. Before initiating the full therapeutic protocol, preliminary test spots were performed on small, representative areas. These tests were essential to verify the tolerability of the selected settings and to exclude any adverse reactions or excessive thermal responses.

For the removal of exogenous particle dyschromia (likely embedded asphalt or soil), a nonablative Nd:YAG 1064 nm laser (Discovery Pico fractional laser, Quanta System SpA) was selected, using the PS handpiece with a 3 mm spot diameter, fluence ranging from 1.5 to 2.0 J/cm², frequency ranging from 1 to 5 Hz, one pass per session, and three treatments performed at intervals of eight to 10 weeks.

The ultrashort pulse duration of the pico laser creates a powerful photoacoustic effect, mechanically shattering deeply lodged particles into minute fragments that can be effectively cleared by the body’s immune system, thereby achieving pigment clearance with minimal thermal injury. Finally, scars in the periorbital area were addressed using a fractional nonablative Nd:YAG 1064 nm laser (Discovery Pico fractional laser, Quanta System SpA), with an 8 mm spot diameter, fluence ranging from 0.5 to 0.8 J/cm², frequency of 5 Hz, one to two passes per session, and three treatments performed at intervals of six to eight weeks.

This gentle yet effective approach was selected to safely induce dermal regeneration via laser-induced optical breakdowns. By remaining nonablative, it offered the lowest risk profile for the exceptionally thin skin adjacent to the orbital reconstruction, successfully stimulating scar revision while maintaining maximum patient safety. This protocol, therefore, represents a comprehensive approach that systematically treats both surface defects and deep structural damage using a sequenced combination of high-impact ablative therapy, safer nonablative consolidation, and targeted regenerative support.

Outcome and clinical observations

Clinical tattoo clearance efficacy was assessed by the physician by comparing baseline and follow-up photographs using a 4-point scale (1, 0-25%, poor; 2, 26-50%, fair; 3, 51-75%, good; and 4, 76-100%, excellent). An objective clinical evaluation was performed at baseline and post-treatment using the Vancouver Scar Scale (VSS) [5]. This scale, which assesses pigmentation (0-2), vascularity (0-3), pliability (0-5), and height (0-3), was implemented to quantify scar improvement for each treated zone.

**Figure 1:**
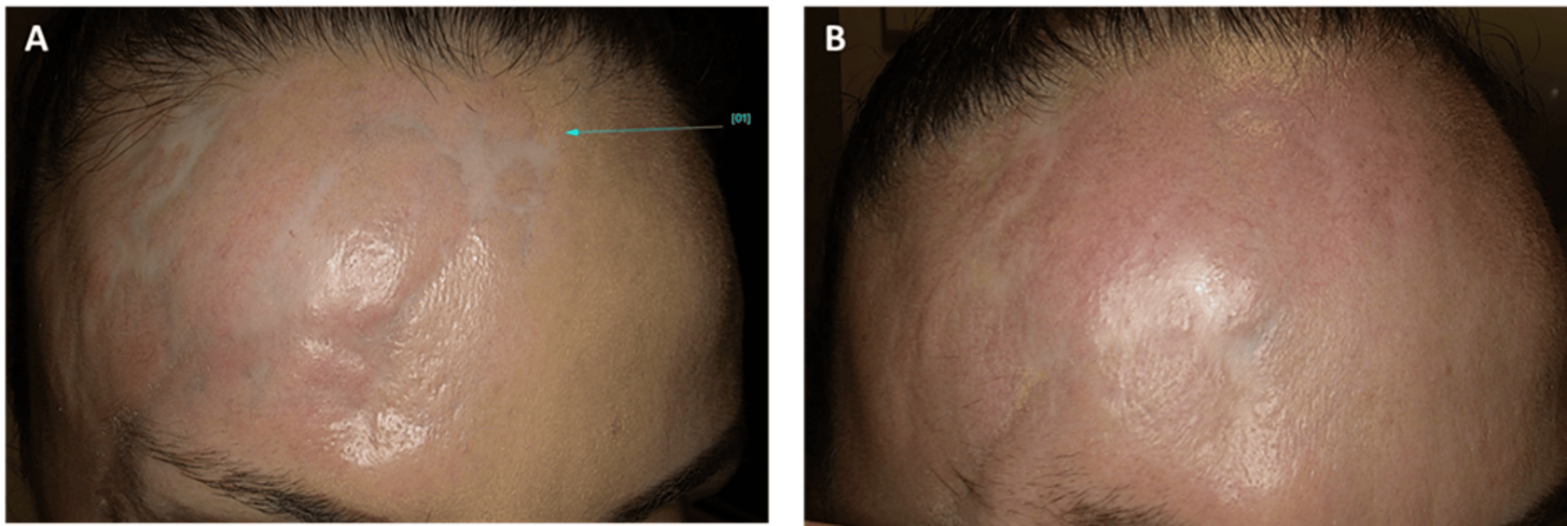
Traumatic scar on the forehead (A) Initial clinical presentation. (B) Post-treatment result at six-month follow-up. The scar was smoothed after completion of all treatment sessions.

The forehead was relatively straightforward. Immediately following the procedure, the patient experienced a burning sensation on the day of treatment, which then evolved into redness lasting approximately four to five days. The outcome was positive, with blurring of the scar structure and a general improvement in skin tone (Figure 1). The baseline VSS score was 6 out of a maximum of 13. At follow-up, a consistent reduction was recorded, culminating in a score of 3/13 (Table 2).

**Table 2:** VSS assessment VSS, Vancouver Scar Scale Source: [5]

Parameter			Forehead area		Right eye area	
			Baseline	Six-month follow-up	Baseline	Six-month follow-up
Pigmentation (0-2)	Normal	0	1	0	2	0.5
	Hypopigmentation	1				
	Hyperpigmentation	2				
Vascularity (0-3)	Normal	0	0	0	2	1
	Pink	1				
	Red	2				
	Purple	3				
Pliability (0-5)	Normal	0	5	3	1	1
	Supple	1				
	Yielding	2				
	Firm	3				
	Banding	4				
	Contracture	5				
Height (0-3)	Normal (flat)	0	0	0	1	1
	0-2 mm	1				
	2-5 mm	2				
	<5mm	3				
Total			6	3	6	3.5

The right eye area was more complex, particularly because of the presence of a titanium plate beneath the skin, which carried the risk of overheating and potential ocular injury. The initial phase (treatment with the PS handpiece) was the most intense. The patient experienced severe erythema lasting seven to 10 days, marked swelling for three to four days, and a burning sensation on the day of treatment. Discomfort was understandably greater due to the presence of the metal plate.

**Figure 2:**
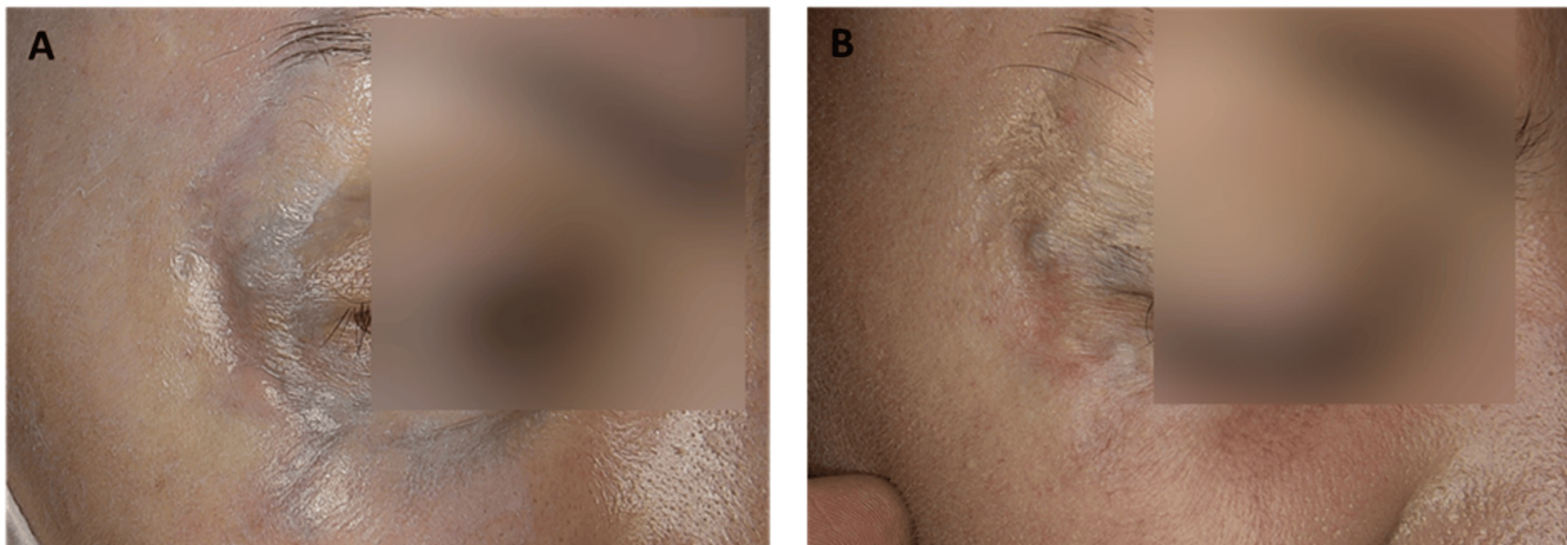
TT (A) Baseline facial profile of the right eye area. (B) Post-treatment result. Excellent tattoo clearance achieved after six months. The remaining blue tint is not residual debris but rather the titanium plate screws shining through the skin. TT, traumatic tattoo

The second fractional treatment phase was considerably milder. The area exhibited redness lasting four to five days, a burning sensation on the day of the procedure, and slight swelling that resolved within one to two days. The final results for the right eye area were significant. After six months of follow-up, blurring of the scar structure and improvement in color were achieved. An interesting observation was that following treatment, the removal of foreign bodies and the appearance of visible punctate bruising highlighted a screw still protruding from the bone, which secures the underlying titanium plate (Figure 2).

The baseline VSS score for this area was 6 out of a maximum of 13. At follow-up, a consistent reduction was recorded, culminating in a score of 3.5/13. The patient achieved excellent clearance of the traumatic asphalt pigmentation, with approximately 90% resolution (Table 2).

Post-treatment care

The post-treatment care strategy varied slightly depending on the treatment area and the intensity of the reaction. For the less complicated forehead area and the milder fractional treatment around the eye, the standard protocol involved the application of ice packs to reduce immediate swelling and temperature, along with regular use of emollients to soothe and hydrate the skin barrier.

However, the initial, more aggressive treatment of the right eye area with the PS handpiece required a more intensive regimen due to severe erythema and swelling, exacerbated by the underlying titanium plate. In addition to ice packs and emollients, the patient in this instance was treated with a 1% hydrocortisone cream for a short duration (four days) to manage inflammation. Notably, no systemic (oral) medications were required to control the patient’s discomfort.

## Discussion

TTs occur when foreign material is embedded in the skin following a puncture, abrasion, or explosion. The most effective initial treatment has historically been the swift and thorough removal of foreign pigmented matter before the wound fully heals. If this initial removal is insufficient, traditional methods to target the remaining particles, such as dermabrasion, cryosurgery, surgical removal, and electrosurgery, invariably lead to scarring or discoloration.

In this challenging scenario, laser removal is considered the most effective method for clearing tattoos. Modern laser treatments are based on the theory of selective photothermolysis, described by Anderson and Parrish in the early 1980s [6]. This theory states that, for selective destruction, the target chromophore must be heated rapidly for a duration no longer than its thermal relaxation time (TRT). The laser energy absorbed by the target chromophore is converted into heat, leading to its destruction. The destroyed particles are subsequently removed by phagocytes and eliminated through transepidermal and/or lymphatic transport.

While the use of QS lasers for cosmetic tattoo removal is widely documented, specific literature on TT removal remains limited. In general, QS lasers seem to be a good choice for delayed removal of TTs, providing reliable clearance with minimal complications.

The literature reports successful monotherapy experiences with the QS ruby laser, often resulting in good clearance but sometimes associated with transient or long-term hypopigmentation, though generally without scar formation [7]. Similarly, studies using the QS alexandrite laser [8] and the QS Nd:YAG laser [9] reported good clearance, typically requiring only a few treatment sessions (averaging 1.7-2.4). However, outcomes are highly variable, with some reports describing good efficacy after a single session [10].

However, in some cases of TTs, the size of the explosive particles may be too large for complete removal. Moreover, QS laser treatment carries a significant risk of triggering secondary micro-explosions of the pigment particles. This process can drive the pigment deeper into the skin and exacerbate surrounding thermal damage, thereby increasing the likelihood of scar formation [1].

Recently adopted PS laser technology addresses this critical risk by leveraging its extremely short pulse duration (in the picosecond range, 10⁻¹² s). PS lasers operate with a dominant photomechanical effect; by delivering energy in pulses much shorter than the pigment’s TRT, the laser instantaneously shatters the pigment into smaller fragments before heat can significantly dissipate into the surrounding tissue. This rapid, targeted action minimizes collateral thermal damage and, crucially, limits the risk of triggering secondary explosions that cause deeper pigment penetration and scarring, offering improved clearance and a lower incidence of side effects such as blistering and pruritus [11,12].

The variability in treatment response reported in the literature for both picosecond and nanosecond lasers is partly linked to the fact that TTs are complex lesions to manage, often requiring patient-specific treatment protocols [11]. Multiple variables influence treatment success, including the nature of the fragments [13-15], depth of penetration, degree of scarring surrounding the fragment, and the patient’s skin type [15]. These factors often lead clinicians to consider multimodal approaches to achieve good clearance, minimize treatment time, reduce side effects and pain, and maximize the final clinical outcome.

The presented case was unique due to a TT complicated by scar tissue and the presence of a titanium plate near the right eye. The metallic implant represented a critical risk factor by introducing the possibility of laser-induced thermal overheating, which could significantly increase adverse side effects. Given the lesion’s extreme complexity, a treatment protocol was designed to integrate as effectively as possible with previous treatments (fractional CO₂ laser combined with PRP injections), ultimately leading to good clearance in a limited number of sessions.

In the less sensitive forehead area, scar treatment was performed using a combination of fractional ablative CO₂ laser with PRP, followed by a fractional picosecond nonablative Nd:YAG 1064 nm laser combined with tropocollagen injections. The association of fractional technology with biostimulants has been described as particularly promising for improving scars, especially atrophic ones [16]. The fractional laser creates controlled microablation columns in the skin, stimulating neocollagenesis and scar tissue remodeling. Its benefits are amplified when used with PRP, which releases growth factors that optimize healing and enhance recovery of fibrotic tissue.

Clinical studies comparing alternating treatments with a QS 1064 nm laser and an ablative fractional CO₂ laser found significantly increased tattoo clearance, greater scar improvement, and a decreased risk of scar worsening compared with QS 1064 nm monotherapy [1].

Dyschromia in the periocular region was treated by combining picosecond laser sessions at low fluence and short pulse duration to minimize thermal damage around the titanium plate. This approach required multiple treatment sessions to achieve good clearance without adverse effects. The clinical outcome demonstrated distinct regional responses. The forehead showed an optimal response, with a marked VSS score reduction reflecting nearly complete scar integration.

In the periocular region, the challenge of exogenous pigment was met with an excellent clearance rate, alongside a corresponding improvement in VSS scores. While the metallic implant complicated the procedure, resulting in exacerbated erythema and swelling lasting seven to 10 days, the final outcome validated the intensive management strategy for complex, pigment-laden scar tissue. This observation strongly underscores the necessity for meticulous parameter selection and intensive post-treatment management, including the use of topical hydrocortisone, in patients with underlying metallic structures.

Limitations

As a case report, the study’s primary limitations are inherent, namely, the limited number of enrolled patients (N = 1) and the lack of a long-term follow-up period. These factors restrict the generalizability of the findings regarding overall safety and efficacy. Nonetheless, this study provides valuable data on an effective and safe alternating laser treatment protocol for managing a black and blue TT complicated by scarring and the presence of a metallic implant.

## Conclusions

Optimal management of complex, scarred TTs necessitates a multimodal, sequential protocol that combines the synergistic benefits of ablative resurfacing, biostimulatory agents, and advanced picosecond technology. In this specific case, the Nd:YAG 1064 nm combination therapy led to appreciable clinical regression and a favorable cosmetic outcome, with near-complete clearance of the lesion at follow-up.
